# Stretchable, Flexible, Breathable, Self-Adhesive Epidermal Hand sEMG Sensor System

**DOI:** 10.3390/bioengineering11020146

**Published:** 2024-02-01

**Authors:** Kerong Yang, Senhao Zhang, Xuhui Hu, Jiuqiang Li, Yingying Zhang, Yao Tong, Hongbo Yang, Kai Guo

**Affiliations:** 1 Division of Life Sciences and Medicine, School of Biomedical Engineering (Suzhou), University of Science and Technology of China, Hefei 230022, China; 2 Suzhou Institute of Biomedical Engineering and Technology, Chinese Academy of Sciences, Suzhou 215011, China

**Keywords:** epidermal sEMG sensor, stretchable structure design, sensor fabrication design, hand function rehabilitation

## Abstract

Hand function rehabilitation training typically requires monitoring the activation status of muscles directly related to hand function. However, due to factors such as the small surface area for hand-back electrode placement and significant skin deformation, the continuous real-time monitoring of high-quality surface electromyographic (sEMG) signals on the hand-back skin still poses significant challenges. We report a stretchable, flexible, breathable, and self-adhesive epidermal sEMG sensor system. The optimized serpentine structure exhibits a sufficient stretchability and filling ratio, enabling the high-quality monitoring of signals. The carving design minimizes the distribution of connecting wires, providing more space for electrode reservation. The low-cost fabrication design, combined with the cauterization design, facilitates large-scale production. Integrated with customized wireless data acquisition hardware, it demonstrates the real-time multi-channel sEMG monitoring capability for muscle activation during hand function rehabilitation actions. The sensor provides a new tool for monitoring hand function rehabilitation treatments, assessing rehabilitation outcomes, and researching areas such as prosthetic control.

## 1. Introduction

Surface electromyographic (sEMG) signals represent a widely employed non-invasive method for recording neural-muscular activity and find frequent application in muscle function assessment [1,2], rehabilitation training [3,4,5,6,7], and human–machine interaction [8,9,10,11]. The advantages of sEMG are mainly reflected in its non-invasive, real-time, and rich information provision. As a non-invasive method, sEMG can capture electrical signals caused by muscle activity by attaching electrodes to the surface of human skin. It has become an ideal tool for understanding muscle activity, reducing discomfort and risk for monitored individuals. In the realm of rehabilitation, sEMG serves as a useful tool for physicians to monitor and assess the activity status and functionality of muscles during the recovery process of rehabilitation patients, facilitating the evaluation of muscle contraction strength [1,12,13,14], degree of muscle fatigue [13,15,16,17], muscle coordination [18,19,20], and other aspects of recovery [21,22]. Particularly in the rehabilitation following neurologic pathway injuries such as stroke [23,24,25] and spinal cord injury [26], the assessment of neuro-muscular pathway recovery at the level of sEMG signals plays a crucial role in evaluating the restorative effects on patients.

In applications such as hand function rehabilitation [27,28,29] and prosthetic control [30], achieving the precise measurement and assessment of finger muscle activity functionality requires collecting ample sEMG signals corresponding to relevant flexor and extensor muscle control. Meattini R et al. proposed a new self-supervised regression human–machine interface (HRi) based on sEMG signals to control wearable rehabilitation robotic arms [27]. They obtained a method with a nonlinear fitting ability by combining non-negative matrix factorization (NMF) with deep neural networks (DNN) and conducted experiments on 10 healthy subjects [27]. Diao Y et al. addressed the issues of poor gesture recognition ability and high abandonment rate in prosthetic systems [30]. Eight healthy adults were recruited, and sEMG data were recorded for seven daily gestures [30]. They proposed an improved fuzzy granular logistic regression (FG-LogR) algorithm for cross-individual gesture classification, which improves the accuracy of cross-individual gesture recognition and has clinical application potential [30]. 

However, traditional surface electromyography collection techniques also have certain limitations. Traditional sEMG sensors mostly use Ag/AgCl electrodes, which often struggle to maintain stable skin contact during dynamic movements. This may lead to signal distortion or instability, affecting the accuracy and reliability of the signal. Meanwhile, traditional electromyographic sensors can cause discomfort or pressure when worn for a long time. In addition, traditional electromyographic sensors are difficult to flexibly adapt to different parts of the human body due to their size, which limits their applicability in specific tasks. Due to the limited surface area available for electrode placement on the dorsum of the hand, standard Ag/AgCl electrodes struggle to form an array-like distribution on the surface. A flexible FPC sEMG sensor array has been proposed to capture sEMG signals in the dorsum of the hand [31,32]. However, the FPC array lacks stretchability, and its modulus significantly differs from that of human skin, leading to failure at the “sensor–skin” interface. The dorsal skin of the hand undergoes substantial deformation during gestures, making it challenging for FPC array electrodes to maintain a stable interface with the skin, resulting in poor signal quality and adversely affecting the assessment outcomes.

Currently, epidermal sensor technologies resembling skin are rapidly advancing for the acquisition of biomedical signals [33,34,35]. These skin-like, flexible, and stretchable electrodes can adapt to the various shapes and sizes of muscles. They possess an elastic modulus similar to the skin and ultra-thin substrate characteristics, enabling conformal contact with the skin [36], thereby exhibiting improved conformity and stability at the “sensor–skin” interface, heightening sensitivity and accuracy in signal acquisition. There are two strategies for constructing epidermal sensors: one is researching inherently stretchable and conductive materials, and the other is conventional brittle inorganic materials with elastomeric substrates patterned via stretchable structures. Metal-patterned flexible sEMG sensors [37,38,39,40] outperform flexible sensors that utilize liquid metals [41], conductive polymers [42], and semiconductor polymers [43] as inherently stretchable and conductive materials, mainly in terms of integration with hardware. Flexible and stretchable electrodes, manufactured from soft and highly biocompatible materials, are designed to minimize irritation and discomfort to the skin, thereby enhancing user comfort [44].

Here, we report a stretchable, flexible, breathable, and self-adhesive epidermal hand sEMG sensor system. The sensor array incorporates circular structures at the intersections of serpentine lines to increase the metal fill factor of the sensors and reduce skin-electrode contact impedance while ensuring sufficient stretchability within a limited sensor placement area, thereby obtaining higher signal-to-noise ratios. Additionally, the carving design is employed to replace the cutting design, effectively reducing the wires’ area. The sensor’s flexible substrate possesses characteristics such as high biocompatibility, low elastic modulus, and breathability, facilitating seamless integration with the skin. Additionally, the sensor is cost-effective and straightforward to manufacture. When combined with our self-made developed acquisition hardware, the entire system weighs approximately 30 g. It enables the real-time detection of high signal-to-noise ratio signals during the hand function rehabilitation-related movements of the fingers and wirelessly transmits them to the host computer.

## 2. Materials and Methods

### 2.1. Fabrication of the Epidermal Hand sEMG Sensors

*(i) Laser patterned cutting of metal copper foil*

Firstly, a mixture of base and curing agent in a weight ratio of 20:1 for polydimethylsiloxane (PDMS) precursor (Dow Chemical Company, Orlando, FL, USA) was spin-coated at 500 rpm for the 20 s on a 100 × 100 mm transparent glass plate (GuLuo^TM^, Luoyang, Henan, China) and cured at 90 °C for 20 min to form the smooth cutting support layer. A layer of copper/polyimide film (DuPont^TM^, Wilmington, NC, USA) was laminated onto the support layer surface. Utilizing a designed CAD file, the copper structures were patterned by a 355 nm UV laser (Delong corporation, Suzhou, China) at a pulse frequency of 50 kHz and a speed of 300 mm/s through a total of 3 times repeated cutting. Nonfunctional materials besides the electrode patterns were removed by delaminating from the PDMS surface, forming the electrodes.

*(ii) Fabrication of flexible substrate*

Laminate a layer of low-viscosity polyethylene terephthalate (PET) film (DuPont^TM^, Wilmington, NC, USA) onto a 100 × 100 mm transparent glass plate (GuLuo^TM^, Luoyang, Henan, China). A mixture of Ecoflex-0030 elastomer (Smooth-On, Macungie, PA, USA) in a weight ratio of 1:1 (A:B) was spin-coated at 500 rpm for 20 s on the PET layer and cured at room temperature for 1 h. Part A and part B of Silbione Gel 4317 (Elkem^TM^, Moon Township, PA, USA) were mixed with a 1:1 weight ratio, and the uncured Silbione Gel 4317 mixture was spin-coated at 500 rpm for 20 s on the cured Ecoflex-0030 layer. It was then cured at 120 °C for 20 min. Finally, the 50 W-CO_2_ laser was used to fabricate micro-holes with raster mode at a power of 6.4 W and a speed of 40.89 mm/s to form the breathable flexible substrate.

*(iii) Transfer printing of electrodes*

The electrodes were picked up from the cutting support layer using water-soluble tape (Aquasol^TM^, Malaga, WA, Australia) and transferred onto the flexible substrate. After dissolving the water-soluble tape with water for 5 min, the epidermal hand sEMG sensor was obtained.

### 2.2. FEA of Mechanical Properties

We employed the commercial software ABAQUS (ABAQUS Analysis User Manual 2010, version 6.10) to the determination of strain distribution (ε) in the stretchable copper foil structure of the sensor under tensile and bending deformations. Both the flexible base layer and copper foil electrodes of the sensor were modeled using tetrahedral elements (C3D10). The model consisted of approximately 2 × 10^4^ elements and the mesh refinement was carried out to ensure convergence. The material properties of the electrodes were specified as Cu, with an elastic modulus (E) and Poisson’s ratio (ν) of $E_{Cu}=119\;GPa$ and $\nu_{Cu}=0.34$, respectively. The flexible base layer was modeled using Ecoflex-0030, employing an isotropic Mooney–Rivlin material model with parameters C10/C01/D1=0.008054/0.002013/2.

### 2.3. Characterizations of Electrical Properties

Pairs of epidermal electrodes and pairs of standard Ag/AgCl electrodes were positioned on the forearm with a 50 mm separation for signal-to-noise ratio (SNR) testing. The signal collection was conducted using PowerLab (ChenHua, Shanghai, China) while subjects simultaneously performed specific tasks, such as gripping with forces of 10/20/40 kg.

### 2.4. In Vitro Evaluation of Cell Biocompatibility

We cultured A549 cells in serum-free medium (RPMI-1640, Kennett Square, PA, USA) supplemented with sterile-filtered L-glutamine, 100 units/mL penicillin, 100 μg/mL streptomycin (Penicillin Streptomycin, South Logan, UT, USA), and 0.1 g of the flexible substrate. The cultivation was carried out at 37 °C in an environment with 5% ${CO}_{2}$ humidity. Subsequently, cell viability was observed under a microscope at 0, 18, and 24 h.

## 3. Result

### 3.1. Design for Epidermal Hand sEMG Sensor System

**Figure 1:**
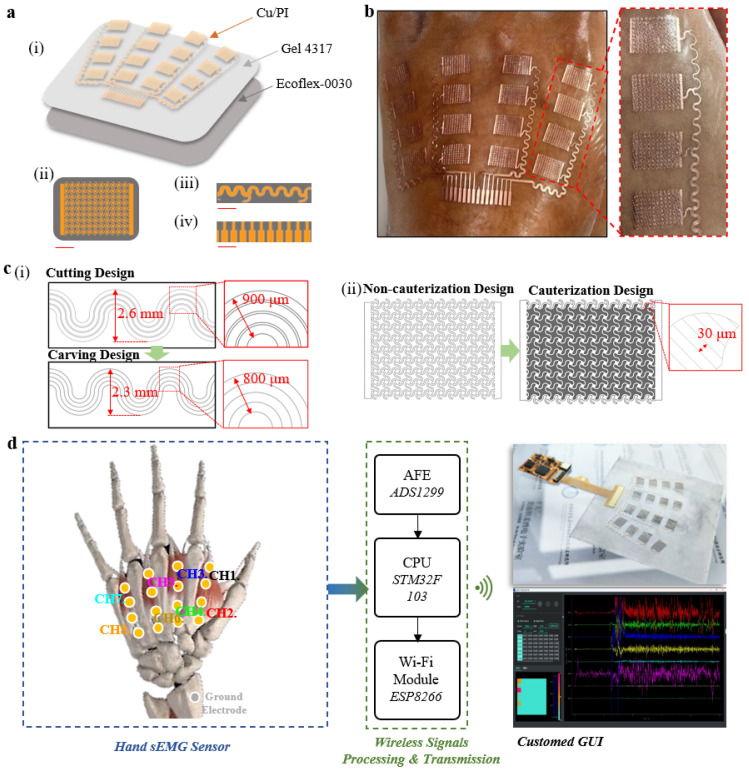
**Overview of the epidermal hand sEMG sensor system.** (**a**) (i) Schematic illustration showing the three functional layers of the sensor. Diagrams of the (ii) optimized stretchable serpentine structure for the sEMG electrode (scale bar, 2.5 mm), (iii) the stretchable metal wires (scale bar, 4 mm), and (iv) cables (scale bar, 3 mm). (**b**) The photograph of the epidermal hand sEMG sensor illustrating the comfortable and soft characteristics when directly attached to the skin without the use of any additional adhesives. (**c**) (i) Diagram illustrating the discrepancies in connect wire layout between cutting design and carving design. (ii) Comparative schematic illustration of single electrode patterns between cauterization design and non-cauterization design. (**d**) The system block diagram illustrating the processing and wireless transmission of signals collected by the sensor.

To determine the parameters of the serpentine lines, circular structures, dimensions of individual electrodes, and distribution parameters of the electrode array for the hand sEMG sensor, we measured the corresponding positions and sizes of muscles, such as the lumbricalis and interosseous dorsalis, involved in hand function rehabilitation training on the palm of a normal adult hand. Considering processing cost and precision limitations, we established the following parameters: a serpentine line width of 100 μm, a serpentine line radius of 100 μm, a circular structure radius of 150 μm, individual electrode dimensions of a 10 mm length and 7 mm width, a metal connect wire width of 200 μm, and a maximum height of the metal connect wires of 2.3 mm. The overall design of the sensor is a fan-shaped sensor array, arranged along the relevant muscles in a configuration of four rows and four columns (Figure 1a).

The hand sEMG sensor consists of three parts: a stretchable conductive metal film layer (Cu/PI, 9 μm/10 μm), an adhesive layer (Silbione RT Gel 4317, Elkem), and an ultra-thin elastic substrate layer (Ecoflex-0030) (Figure 1a,i). The ultra-thin conductive metal film layer (Cu/PI, 9 μm/10 μm) was chosen for its excellent conductivity, mechanical strength, and biocompatibility. Silbione RT Gel 4317, with high adhesion strength and an extremely low elastic modulus, ensures a tight bond between the elastic substrate layer and the metal film layer while maintaining a conformal adhesive contact state between the metal film layer and the skin. Ecoflex-0030 was selected for its low elastic modulus, stretchability, biocompatibility, and ease of demolding. To address the relatively low metal fill factor in stretchable serpentine sensors [37,38,39,40], we designed a stretchable structure. While ensuring the stretchability of the circuit with serpentine lines, circular structures were introduced at the intersections of the serpentine lines to enhance the metal fill factor within the electrode area, improving signal quality (Figure 1a,ii). Individual electrodes are connected to the cable via serpentine connect wires (Figure 1a,iii). The 10-pin flexible cables (Figure 1a,iv) provide the sensor with the capability to integrate external data acquisition and wireless transmission hardware. The stretchable design and multi-layered material strategy of the hand sEMG sensor allow for direct application to the skin, eliminating the need for additional adhesives (Figure 1b).

To address the challenge of limited surface area for electrode placement on the dorsum of the hand and to efficiently arrange a feasible number and area of the electrodes within these constraints, we propose a carving design with laser cutting. The carving design uses cut-off rather than excision (used in the cutting design) of the portion between the connecting wires to minimize the distance before the neighboring connecting wires without affecting the connecting wire line width (200 um). Compared with the cutting design, this design effectively reduces the spatial distribution of the trace, minimizing the maximum span length from 2.6 mm to 2.3 mm and reducing the maximum summarized trace width from 900 μm to 800 μm, allocating more space to the electrodes (Figure 1c,i). To reduce the manual manufacturing cost of the electrodes, we report a cauterization design as a replacement for the cutting design (Figure 1c,ii). The cauterization design refers to the design of laser cauterization lines between the stretchable structures inside the electrodes and the design of the cauterization line distance in conjunction with the UV laser (LM-UV-3, DeLong, Suzhou, China) cauterization line width (~30 μm). Compared with the non-cauterization design, the cauterization design reduces the time cost (from ~3 h to ~45 min), and labor cost (from requiring manual etching of the internal microstructure to not requiring manual etching of the internal microstructure) and has a high repeatability (production success rate increasing from ~20% to 100%) in large-scale production.

We present a system design strategy (Figure 1d) for processing the electrical signal flow in sEMG monitoring. A standard Ag/AgCl electrode used as the ground electrode contributes to the enhancement of the common mode rejection ratio (CMRR) for acquiring high-quality signals. The signal is first amplified and filtered by the analog front-end (AFE) chip (ADS1299-8, TI). Subsequently, the built-in analog-to-digital converter (ADC) converts the analog signal to voltage at a sampling rate of 500 Hz. The collected data are then processed by the microcontroller unit (MCU) chip (STM32F103) and wirelessly transmitted via Wi-Fi (ESP8266) for further analysis on user devices. Finally, our custom-designed graphical user interface (GUI) is integrated to display and record real-time sEMG waveforms and data. In addition, an adjustable bandpass filter has been added to further eliminate noise. The integration of stretchable and flexible sensors with wireless acquisition hardware provides a convenient and accurate new solution for the direct collection of sEMG signals related to finger actions.

### 3.2. Fabrication and Wearing of Epidermal Hand sEMG Sensors

**Figure 2:**
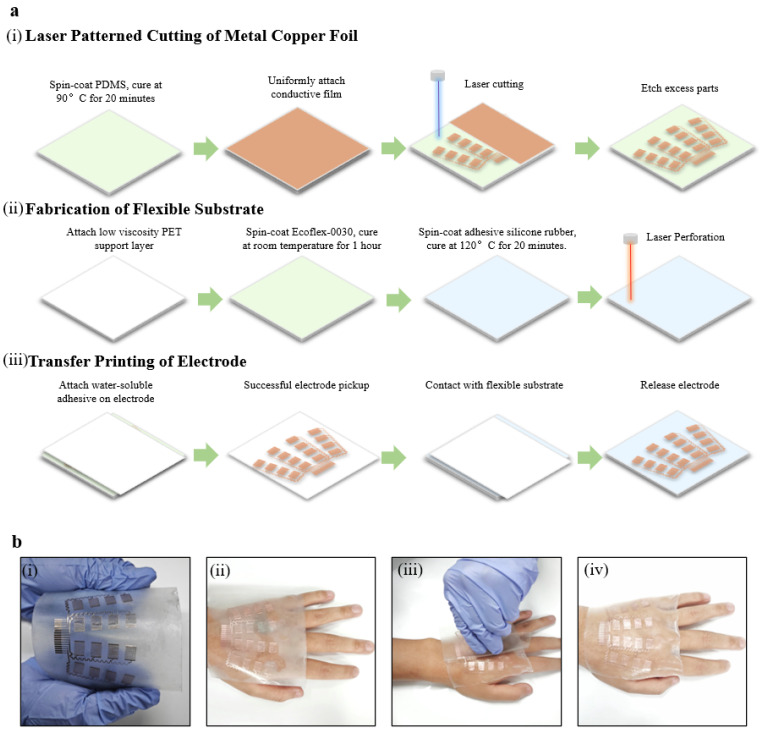
**Illustration of the fabrication and wearing of the epidermal hand sEMG sensor.** (**a**) Flowchart of the sensor fabrication, including (i) laser-patterned cutting of metal copper foil, (ii) fabrication of flexible substrate, and (iii) transfer printing of electrodes. (**b**) Flowchart of the sensor wearing processes.

As shown in Figure 2a, the fabrication of the epidermal hand sEMG sensor can be divided into three main steps: (i) the laser-patterned cutting of the metal copper foil, (ii) the fabrication of the flexible substrate, and (iii) the transfer printing of electrodes. During the laser-patterned cutting of metal copper foil, a layer of polydimethylsiloxane (PDMS) mixture is spin-coated onto a clean glass plate and cured to provide a smooth cutting support layer. Subsequently, a layer of metal copper foil is laminated onto the cured cutting support layer, forming a closely connected interface through van der Waals forces. Next, the metal copper foil is patterned using a UV laser (LM-UV-3, DeLong), with cauterization cutting applied to the interior of individual electrodes and carving cutting applied between metal connection wires. Finally, the redundant portions are physically removed, forming the electrodes (Figure 2a,i). The flexible and transparent substrate is obtained by spin-coating and curing Ecoflex-0030 and Gel 4317 in turn on a hard support layer surface laminated to a clean glass plate. A CO_2_ laser (VLS 3.50, Universal, Newark, DE, USA) is then applied to introduce micro-holes, providing the flexible substrate with additional breathability for enhanced comfort and skin friendliness during wearing (Figure 2a,ii). Ultimately, the electrodes are picked up from the cutting support layer using water-soluble tapes and laminated onto the flexible substrate. After deactivating the water-soluble tapes, the electrodes are released intact onto the flexible substrate. The adhesive force on the substrate surface ensures a secure bond between the electrodes and the flexible substrate, forming the epidermal hand sEMG sensor (Figure 2a,iii). The fabrication of the epidermal hand sEMG sensor is simple, cost-effective, and suitable for large-scale industrial production.

The application of the epidermal hand sEMG sensor is straightforward and convenient. Users gently press the sensor onto the skin, following the direction of the hand muscles. The demolding agent on the hard support layer, the easy demolding properties of Ecoflex-0030, and the adhesive properties of Gel 4317 ensure that the sensor can be easily separated from the support layer, and it tightly adheres to human skin (Figure 2b). A simple one-piece attachment operation helps to reduce the time and complexity for physicians or users, as well as labor costs.

### 3.3. Mechanical, Electrical, and Biocompatibility Performance of Epidermal Hand sEMG Sensors

**Figure 3:**
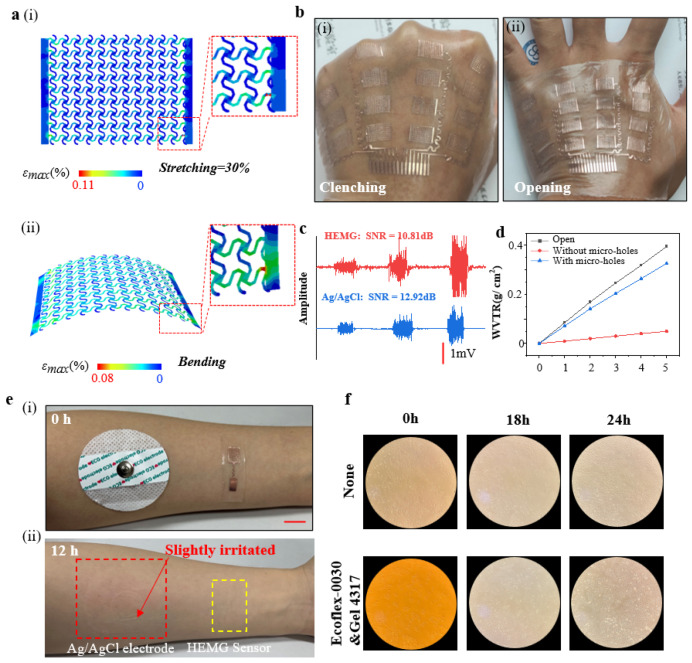
**Mechanical, electrical, and biocompatibility performance of epidermal hand sEMG sensors.** (**a**) FEA result diagrams of electrode strain distribution, (i) induced by 30% uniaxial stretching in the transverse direction, and (ii) induced by bending strain. (**b**) Photos of the sensor in contact with the skin during various mechanical deformations such as clenching and opening, (i) Clenching hand, (ii) Opening hand. (**c**) Measurement of EMG signals using the electrodes (red) and standard Ag/AgCl electrodes (blue). (**d**) Water vapor transmission range of the flexible substrate with and without micro-holes, and bottle opening. (**e**) Photographs of the forearm after detachment of the standard Ag/AgCl electrode and the epidermal electrode 12 h later, demonstrating no skin irritation with the epidermal sEMG electrode. (i) Just put on the sensor, (ii) removing the sensor after 12 h. (**f**) Optical microscope images of human lung fibroblast cells cultured for 0, 18, and 24 h on Ecoflex-0030&Gel 4317.

The optimized electrode exhibits sufficient stretchability and flexibility. When subjected to uniaxial stretching up to a maximum skin strain of 30% [45], the maximum strain in the copper foil of the electrode is 0.11%, significantly lower than the yield strain of Cu (0.3%) [46], as confirmed by a finite element analysis (FEA) (Figure 3a,i). Additionally, the FEA of bending for the electrode shows a maximum strain in the copper foil of 0.08%, also below the yield strain of Cu (Figure 3a,ii). Subsequently, when the sensor has adhered to the dorsum of the hand, it is capable of withstanding mechanical deformations of the skin, such as clenching and opening (Figure 3b).

In the case where the electrode dimensions (length 10 mm, width 7 mm) are 1/5 of the standard Ag/AgCl electrode dimensions (radius ~52 mm), the electrode demonstrates a comparable signal-to-noise ratio (~10.81 dB) when compared to the standard Ag/AgCl electrode (~12.92 dB) (Figure 3c). Furthermore, the flexible substrate, treated with micro-holes using a CO_2_ laser, exhibits excellent breathability. The water vapor transmission rate (WVTR) of the flexible substrate with micro-holes (~2.719 mg cm^−2^ h^−1^) is over six times that of the substrate without micro-holes (~0.412 mg cm^−2^ h^−1^), comparable to an open bottle (~3.295 mg cm^−2^ h^−1^) (Figure 3d).

Compared to the standard Ag/AgCl electrode, this electrode does not induce additional skin irritation or allergic reactions after being adhered to the skin for 12 h (Figure 3d). Furthermore, we conducted cytotoxicity tests on the substrate material. We cultured human lung adenocarcinoma cells (A549) on the flexible substrate materials (Ecoflex-0030 and Gel 4317) for 24 h, and the results indicate that cell growth is unaffected, demonstrating the biocompatibility of the flexible substrate materials (Figure 3f).

### 3.4. Signal Acquisition Applications of Epidermal Hand sEMG Sensors

**Figure 4:**
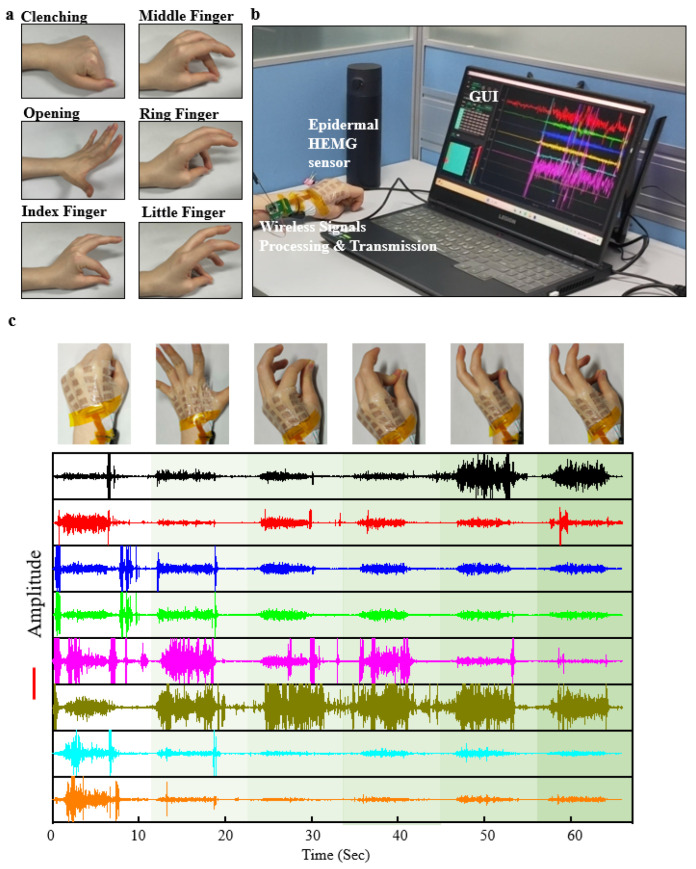
Demonstration of signal acquisition applications for epidermal hand sEMG sensors. (**a**) Schematic representations of common hand function rehabilitation movements. (**b**) Photograph illustrating the connection between the epidermal hand sEMG sensor system and the mainframe during signal acquisition. (**c**) Eight-channel sEMG signals recorded during six different hand function rehabilitation movements.

Hand function rehabilitation is a process that employs various therapeutic interventions to train the muscles and nervous system of the hand, aiming to promote hand function recovery and enhance the quality of life for patients [47,48]. Common hand rehabilitation actions include clenching, opening, and finger opposition (Figure 4a). The rehabilitation training of these actions is a crucial component of hand function rehabilitation, especially for patients with impaired finger function due to trauma, neurological disorders, hand diseases, or other causes. The epidermal hand sEMG sensor can capture signals from the hand, which are wirelessly transmitted in real-time to the GUI interface for visualization and data recording, facilitated by the integrated wireless processing hardware (Figure 4b). Each movement by the subject triggers’ distinct patterns of EMG signals (Figure 4c), demonstrating the sensor’s capability to real-time capture muscle EMG signals related to hand function rehabilitation movements. We believe that in the future, the epidermal hand sEMG system holds significant potential for use in more refined applications such as monitoring hand function rehabilitation treatments, assessing rehabilitation outcomes, prosthetic control, and beyond.

## 4. Discussion

The new epidermal sEMG sensor developed in this article for the back of the hand has the following characteristics and advantages.

(1) The sensor adopts a circular structure design with stretchability, flexibility, and a high metal filling rate. This improves the scalability and signal acquisition ability of the electromyographic sensor. The process design of laser engraving ensures the maximum electrode reserve size, further optimizing the performance of the sensor.
(2) The sensor adopts a multi-layer strategy combined with a low modulus, high viscosity, and biocompatible materials. At the same time, the CO_2_ laser micro-holes treatment allows the sensor to seamlessly and breathably combine with the skin, avoiding irritation and allergic reactions to the skin. These characteristics help optimize the signal-to-noise ratio of sensors, improve signal quality, and maintain user comfort.
(3) The sensors and their manufacturing processes proposed in this article are lower cost, and the cauterization design reduces labor costs and improves the fabrication success rate, which makes it easy to manufacture on a large scale, thus promoting the possibility of their widespread application.

The experimental verification of the article shows that the flexible electromyographic sensor developed in this paper can capture real-time muscle surface electromyographic signals related to hand function rehabilitation movements. This provides new tools and possibilities for monitoring rehabilitation progress, evaluating treatment outcomes, and researching prosthetic control.

The next step of the research will focus on the clinical hand function rehabilitation assessment and training application. In detail, for hand function rehabilitation assessment, the next step of the research is to collect more sEMG signals of the unaffected hand and the affected hand of hand dysfunction patients at different rehabilitation stages, and then build a scientific, quantitative, and systematic hand function rehabilitation assessment system based on artificial intelligence algorithms and physician’s rehabilitation assertions. For hand function rehabilitation training, the next research is using signals from the healthy hand of hand dysfunction patients for pattern recognition model building and ultimately achieving real-time mirroring hand rehabilitation training.

However, there are certain limitations to the research in this article: this article only compares the biocompatibility of traditional Ag/AgCl and the sensor in this article from the perspective of skin indentation after long-term wear on the human body. Before large-scale application, it may be necessary to conduct more biocompatibility testing through third-party monitoring agencies.

## 5. Conclusions

We report a stretchable, flexible, breathable, and self-adhesive epidermal hand sEMG sensor designed for the wireless monitoring of sEMG signals directly from the skin on the back of the hand. The circular structure incorporated between serpentine lines is designed for stretchability, flexibility, and high metal fill ratio. The carving design ensures maximal electrode reserve size. The multi-layered strategy employing low-modulus, high-adhesive, and biocompatible materials, combined with the CO_2_ laser micro-holes treatment, facilitates the seamless and breathable integration of the sensor with the skin, avoiding irritation and allergy, all contributing to an optimal signal-to-noise ratio. The cost-effective manufacturing process, coupled with the cauterization design, makes sensors easy to manufacture on a large scale. Experimental validation demonstrates the sensor’s capability to real-time capture sEMG signals from muscles associated with hand function rehabilitation movements, providing a novel tool for monitoring rehabilitation progress, evaluating treatment outcomes, and prosthetic control research.

## Data Availability

The data that support the findings of this study are available from the corresponding author upon reasonable request.
